# Supplementary material for: From In Silico Simulation between TGF-β Receptors and Quercetin to Clinical Insight of a Medical Device Containing Allium cepa: Its Efficacy and Tolerability on Post-Surgical Scars
Source: Life (Basel). 2023 Aug 21;13(8):1781. doi: 10.3390/life13081781 (PMC10455185; doi:10.3390/life13081781)
Supplement: Supplementary file 1 [file life-13-01781-s001.zip › life-2518265-supplementary.pdf]

Table S1. Vancouver scar scale.

**Scar's charactetistic**		**Score**
Height	Flat	0
	< 2mm	1
	2-5 mm	2
	>5 mm	3
Elasticity	normal	0
	Flexible	1
	Saggy	2
	Immobile	3
	Contracted	4
Pigmentation	normal	0
	Hypopigmented	1
	Hyperpigmented	2
Vascolarization	Normal	0
	Rose	1
	Red	2
	Purple	3
Total score		/12

Table S2. Manchester Scar Scale.

**Scar's charactetistic**		**Score**
Colour	Perfect	1
	Slight mismatch	2
	Obvious mismatch	3
	Gross mismatch	4
Finish	Matter	1
	Shine	2
Contour	Flush with surrounding skin	1
	Indented	2
	Hypertrophic	3
	Keloid	4
Distorsion	None	1
	Mild	2
	Moderate	3
	Severe	4
Texture	Normal	1
	Just palpable	2
	Firm	3
	Hard	4
Total score		/16

Table S3. Stony Brook Scar evaluation scale.

	Scar	Score
Width	>2mm	0
	<2 mm	1
Height	Elevated/depressed	0
	Flat	1
Color	Darker	0
	Surrouding skin	1
Hatch marks or Suture marks	Present	0
	Absent	1
Overall apparence	Poor	0
	Good	1
Total score		/5

Table S4. POSAS scale (Patient and Observer Scar Assessment Scar).

NORMAL SKIN	1	2	3	4	5	6	7	8	9	10	WORST SCAR IMAGINABLE
**Observed scar assessment**											
Vascularity											
Pigmentation											
											Hypo
											Hyper
											Other
Thickness											
Refile											
Pliablity											
Total score observe scar scale											
**Patient scar assessment**											
painful											
Itching											
Colour											
Stiff											
Thickness											
Irregular											
Total score patient scar scale											
